# A Case of Autoimmune Pancreatitis Presenting As Alcohol-Induced Necrotizing Pancreatitis

**DOI:** 10.7759/cureus.39616

**Published:** 2023-05-28

**Authors:** Zachary A Creech, Divya Shastri, Mohammed Wajid Hussain, Waleed Ikram, Mark MacElwee

**Affiliations:** 1 Department of Internal Medicine, Creighton University School of Medicine, Phoenix, USA; 2 Department of Internal Medicine, Valleywise Health Medical Center, Phoenix, USA

**Keywords:** autoimmune pancreatitis, computed tomography abdomen and pelvis, igg-4 related disease, alcohol use disorder, internal medicine and endocrinology, pancreatitis causes, autoimmune pancreatitis (aip)

## Abstract

Autoimmune pancreatitis (AIP) is an inflammatory condition of the pancreas, commonly characterized by elevated levels of immunoglobulin G (IgG) 4. Diagnosis of this condition can be challenging in patients with risk factors for other pancreatitis etiologies and requires a comprehensive approach utilizing clinical, radiologic, and laboratory findings. Here, we present a case of an individual with a history of multiple prior hospitalizations for alcoholic pancreatitis, who presented with symptoms of abdominal pain, nausea, and vomiting. Computed tomography (CT) imaging revealed intra-abdominal abscesses and findings consistent with pancreatitis. Further laboratory results revealed elevated lipase and IgG4 levels, indicating AIP as the underlying cause. This case highlights the importance of considering AIP as a differential diagnosis in individuals presenting with pancreatic disease.

## Introduction

Pancreatitis is an inflammatory condition that leads to autodigestion of the pancreas and is a leading cause of hospitalizations worldwide related to the gastrointestinal tract [1]. Pancreatitis etiologies vary in frequency and severity [1,2]. Acute pancreatitis occurs in approximately 34 people per 100,000, with 21% of patients developing a recurrence and 36% of recurrent acute pancreatitis patients developing chronic pancreatitis [2]. The two most common causes of acute pancreatitis are gallstones and alcohol-induced pancreatitis [1].

Chronic pancreatitis is caused by persistent inflammation of the pancreas, commonly resulting in irreversible pancreatic damage [3]. The incidence of chronic pancreatitis is lower than acute pancreatitis, ranging from five to 12 cases per 100,000 people worldwide, but it can still have a significant impact on a patient’s quality of life [4]. Chronic pancreatitis is also commonly caused by long-term alcohol abuse, particularly in developed countries, although genetic predisposition, obstructive pancreatic disease, and autoimmune disease are additional causes [5]. While autoimmune pancreatitis (AIP) makes up roughly 2% of all chronic pancreatitis cases, this rare etiology is becoming increasingly important as a recognized cause of recurrent acute pancreatitis, chronic abdominal pain, or painless jaundice [6,7].

Here, we present a unique case of an individual with presumed alcohol-induced pancreatitis, presenting with an elevated immunoglobulin G (IgG) 4 level and symptoms indicative of AIP. This case highlights the importance of considering AIP as a differential diagnosis in individuals presenting with pancreatic disease, and particularly those with an elevated serum IgG4 level. We discuss the diagnostic challenges, clinical course, and management of this rare and complex presentation of pancreatic disease.

## Case presentation

A 50-year-old man with a significant past medical history of hypertension, type 2 diabetes, and multiple hospitalizations for alcoholic pancreatitis presented to the emergency department with worsening right lower quadrant abdominal pain accompanied by subjective fevers, chills, and diaphoresis. The patient’s pain was also associated with nausea, abdominal distension, and non-bloody, non-bilious emesis. Review of systems revealed no evidence of recent weight fluctuations, shortness of breath, dysuria, hematuria, lethargy, or rash. The patient's social history was indicative of prolonged alcohol abuse, as he endorsed up to six alcoholic drinks daily. He denied recreational and illicit drug use but admitted to occasional tobacco consumption. Approximately one month before presenting to the emergency department, the patient was hospitalized for spontaneous bacterial peritonitis, necrotizing pancreatitis, and an intra-abdominal abscess that was treated with right flank and suprapubic drain placement, as well as a course of intravenous ceftriaxone and oral metronidazole.

At the time of admission, the patient's vital signs were significant for hypotension, with a blood pressure of 95/65 mmHg. Additional vital signs included a heart rate of 93 beats per minute, a respiratory rate of 18 breaths per minute, oxygen saturation at room air of 100%, and a temperature of 36.9 degrees Celsius. The initial physical examination revealed a swollen, tender abdomen with guarding in all four quadrants, but no signs of peritonitis. Palpation of the right flank resulted in intense abdominal discomfort. Bowel sounds were present in all quadrants, and no jaundice, irregular heart rate, murmur, dyspnea, or consolidations were noted. The patient's suprapubic drain was still in position from his hospitalization one month prior, with turbid yellow to white fluid observed in the drain. Laboratory values were indicative of mild anemia with a hemoglobin level of 9.6 g/dL, lipase level of 2,201 U/L, and thrombocytosis with platelets recorded at 565 K/μL, likely due to the patient’s underlying inflammation. Additional relevant laboratory values at the time of administration are listed in Table 1.

**Table 1:** Significant laboratory values at the time of admission

Variable with Units	Reference	Day of Admission
White blood cell count, K/μL	3.4-11.0	9.1
Neutrophil, %	38.3-74.8	79.5
Hemoglobin, g/dL	11.3 - 16.8	9.6
Platelets, 1000/µL	147 - 395	565
International Normalized Ratio (INR)	0.9-1.1	1.5
Lipase, U/L	23-300	2201
Creatinine, mg/dL	0.60-1.30	.39
Triglycerides, mg/dL	<150	117
Aspartate Aminotransferase (AST), U/L	17 - 59	13
Alanine Aminotransferase (ALT), U/L	4 - 50	5

**Figure 1:**
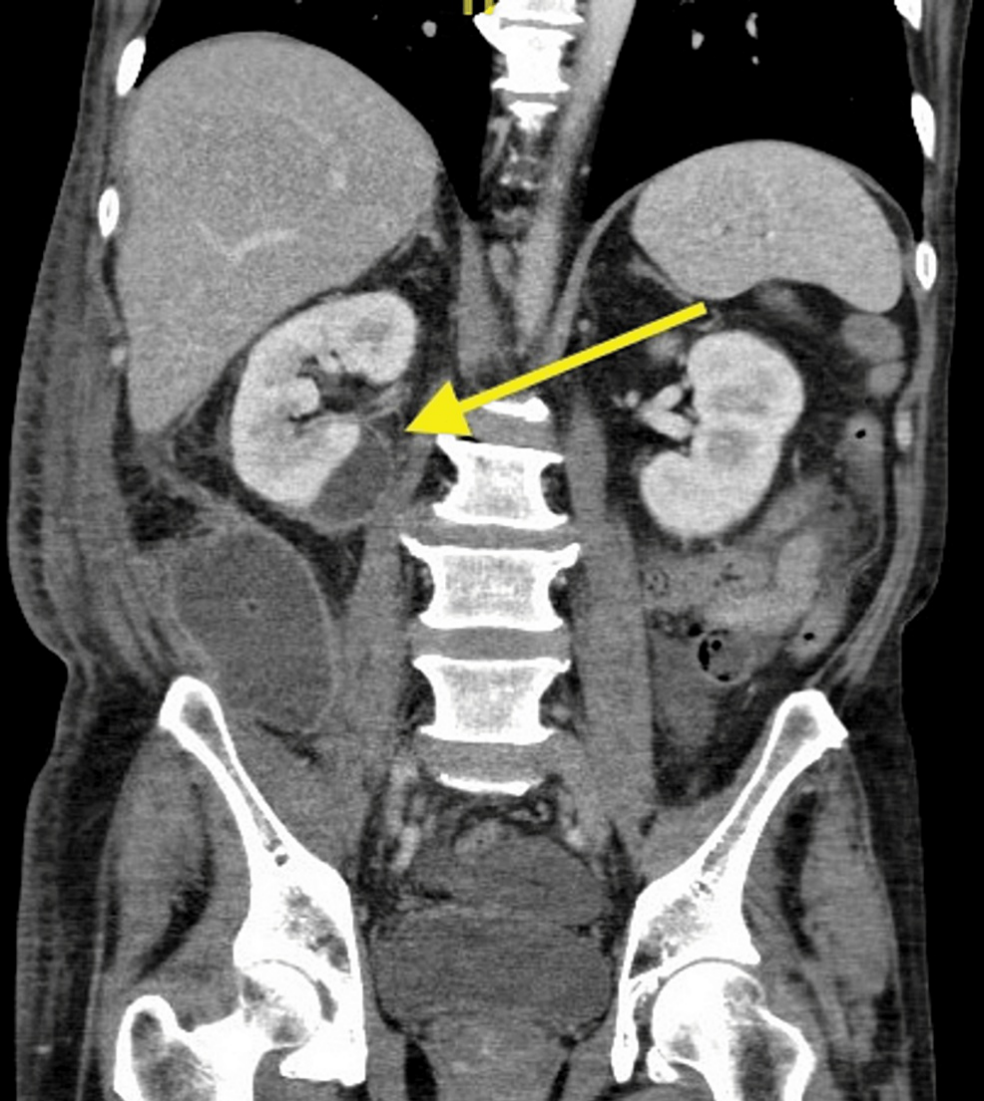
CT of the abdomen and pelvis The yellow arrow depicts a 2 x 6.4 x 4.3 cm abscess and capsular fluid surrounding the right medial inferior pole of the right kidney

**Figure 2:**
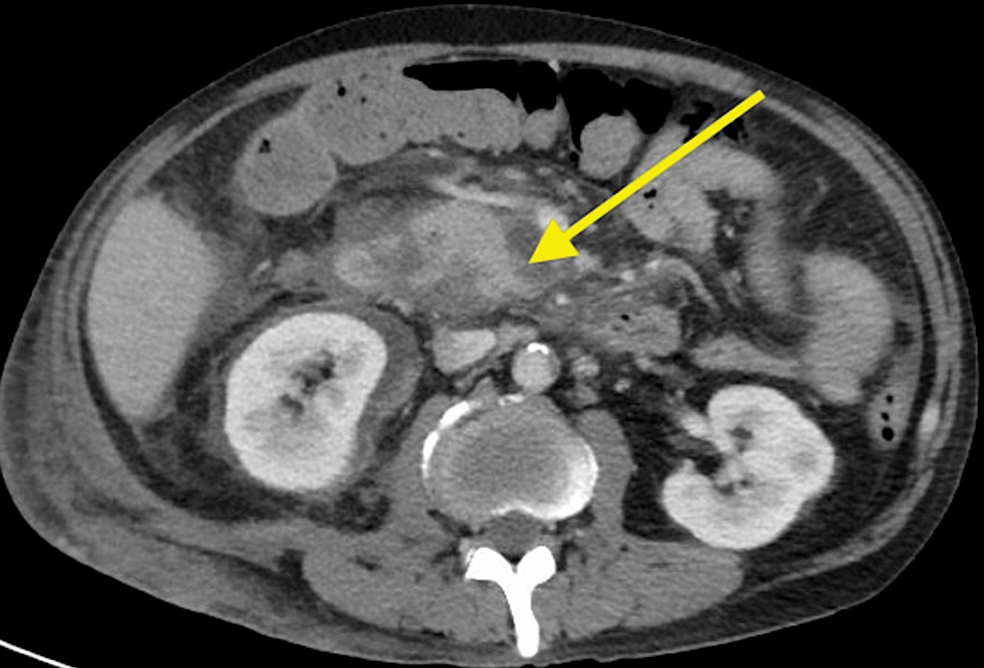
CT of the abdomen and pelvis The yellow arrow depicts the patient’s necrotizing pancreatitis and peripancreatic fluid collections found on CT imaging

Computed tomography (CT) of the abdomen and pelvis revealed an expanding, 2 x 6.4 x 4.3 cm, right medial inferior pole subcapsular renal abscess collection with mass effect on the kidney and a right 5.5 x 5.6 x 8.1 cm posterior pararenal abscess, extending from the posterior pararenal space inferiorly to the pelvis. CT also demonstrated pancreatic head and uncinate necrotizing pancreatitis with increased surrounding perinephric fat stranding in the surrounding area. CT imaging findings are included in Figures 1, 2.

Following the imaging reports, interventional radiology was consulted for drainage of the right perirenal abscess. Ultrasound-guided drainage of the abscess yielded 20 mL of purulent dark brown fluid. Fluid analysis revealed an elevated nucleated cell count and red blood cell count. Given the patient's long-term use of ceftriaxone and metronidazole, as well as the concern for antibiotic resistance, the patient was switched to piperacillin-tazobactam 3.375 g intravenously every eight hours.

Two days after admission, the patient's IgG laboratory levels returned, indicating an elevated IgG4 level of 302 mg/dL (reference range: 1-123 mg/dL), while IgG 1, 2, and 3 were within normal limits. Additional laboratory results revealed a cancer antigen 19-9 level of 14 U/mL (reference range 35 U/mL), which was within normal limits. The patient's findings were highly suggestive of AIP, given the patient's significantly elevated IgG4 levels and abdominal pain, as well as imaging findings consistent with pancreatitis.

Gram stains and cultures of intra-abdominal drainage were negative during the patient's hospitalization. The patient was administered morphine and oxycodone as needed for pain management and was encouraged to feed enterally as tolerated. His pain levels began to decrease steadily throughout the admission and the patient was ultimately stabilized. The patient was administered piperacillin-tazobactam for the duration of his admittance, which was a total of nine days. Prior to discharge, the patient’s suprapubic and right flank drain were removed because drainage output was reduced to less than 10 mL a day. The patient was then discharged to a skilled nursing facility to complete the remaining duration of antibiotics and was advised to begin prednisone 40 mg daily four days after discharge, with an anticipated treatment duration of 40 days. The patient was scheduled to follow-up with his primary care physician, rheumatology, and general surgery to monitor for disease progression, treatment outcomes, and steroid taper, but he was ultimately lost to follow-up.

## Discussion

In this case report, we discuss a patient who initially presented with a suspected case of alcohol-induced pancreatitis, but subsequent laboratory findings were suggestive of AIP. Although AIP and alcohol-induced pancreatitis have distinct pathophysiological mechanisms, they can share similar clinical features, making it challenging to diagnose AIP in patients with risk factors for other pancreatitis etiologies.

First described in 1961 by Sarles et al. [8], AIP is currently classified into two subtypes on the basis of its prominent histological characteristics [8,9]. Type 1, also known as lymphoplasmacytic sclerosing pancreatitis, and type 2, also known as idiopathic duct-centric chronic pancreatitis, are the two predominant subtypes of AIP [10-12]. The International Consensus Diagnostic Criteria was established in 2011 to correctly identify patients with AIP, and their specific subtype [9,10,13]. Cardinal features suggestive of AIP include radiological imaging characteristically showing a localized or diffuse enlargement of the pancreas and parenchyma without pancreatic duct dilation, positive serology consisting of IgG4, IgG, and antinuclear antibody, involvement of other organ systems, characteristic histopathology of the pancreas, and response to steroid therapy [10,13-15], While definitive identification of the specific subtype of AIP does require histological confirmation through biopsy procedures, other features can assist in the diagnosis. Typical serological abnormalities, such as elevated IgG4, are characteristic of type 1 AIP [13]. Both types of AIP are associated with inflammatory bowel disease, but type 2 AIP has more of an association [13]. Response to steroid therapy and imaging features cannot solely distinguish the specific type of AIP but can also aid in making the general diagnosis [13]. Lymphoplasmacytic sclerosing pancreatitis is more commonly reported to involve other organs than type 2 AIP [9,13]. If other organ involvement is present, the biliary system is the most commonly affected, but reports have also noted plasmacytic exocrinopathy of the salivary glands, pulmonary and retroperitoneal fibrosis, interstitial nephritis, and thyroiditis [9]. However, it is important to note that, even in the absence of other organ involvement or an elevated IgG4 level, the diagnosis of type 1 AIP can still be made as some cases of type 1 AIP are seronegative and lack organ involvement outside of the pancreas [13].

AIP can present clinically with nonspecific abdominal pain, weight loss, new-onset diabetes mellitus, recurrent episodes of acute pancreatitis, or painless jaundice [14]. Consistent with AIP, the patient's CT scan in Figure 2. revealed significant edema and inflammation surrounding the pancreas. Peripancreatic fat stranding has been reported in a small subset of confirmed cases of AIP, which was also observed on CT imaging of our patient [15]. However, a pancreas that appears normal on imaging does not rule out AIP [10].

In 2001, Hamano et al. [16] described the use of IgG4 in diagnosing AIP and differentiating it from malignancy and other causes of pancreatitis [13,16,17]. Specifically, serum IgG4 levels greater than 140 mg/dL have been reported to have a sensitivity of 86% and a specificity of up to 96% for diagnosing AIP, and our patient's labs revealed an IgG4 level of 302 mg/dL [18,19]. Although our patient self-reported consuming up to six standard alcoholic drinks per day, his alcohol consumption did not frequently exceed the estimated threshold for alcoholic pancreatitis. It has been estimated that a person must consume 10 to 11 standard United States drinks over a period of six to 12 years in order to develop pancreatitis-like symptoms [20]. 

Early diagnosis and treatment of AIP with high-dose corticosteroids can improve clinical outcomes and reduce the need for unnecessary interventions [18]. The most prescribed dosing and treatment duration for steroid therapy in AIP is prednisolone 0.6 mg/kg/day or prednisone 40 mg once daily for a total of four weeks with a gradual tapering of the medication [18]. The goal of steroid therapy is to provide not only symptomatic relief for the patient but also limit organ dysfunction [18]. Relapse of AIP has been reported in approximately 50% of patients after steroids are discontinued [18]. Regardless of the cause of pancreatitis, the treatment also focuses on nutritional support and pain management. Complications of AIP can include pseudocyst formation or mesenteric vessel thromboses, and as highlighted in our case, peripancreatic collections and necrotizing pancreatitis [18]. 

The complexity of diagnosing autoimmune disease in patients with concurrent alcohol use has been reported in other gastrointestinal conditions as well. In 2021, a retrospective study identified a total of 12 cases in which patients were initially evaluated for alcoholic liver disease but ultimately met the criteria for autoimmune hepatitis [21]. The cohort of patients that met the criteria for autoimmune hepatitis was characterized by increased rates of progression to cirrhosis and hepatic-related deaths [21]. Another case, reported in 2018, detailed an individual with a history of alcohol abuse that presented with elevated antinuclear antibody levels [22]. However, a liver biopsy revealed severe hepatic steatosis more consistent with alcoholic hepatitis rather than an autoimmune disease, despite elevated antinuclear antibody results [22]. These reports represent some of the diagnostic challenges faced by clinicians and highlight the importance of establishing guidelines to differentiate patients that initially present for suspected alcohol-induced disorders but may have underlying autoimmune disease.

## Conclusions

Given the rarity of AIP, a high index of suspicion is necessary for early diagnosis and treatment. Histological samples were not obtained from our patient, but the diagnosis was made based on a combination of clinical, radiologic, and laboratory findings. By presenting this case, we hope to educate providers on the importance of maintaining a broad differential in order to improve prognosis and treatment outcomes for patients afflicted with relatively uncommon conditions, such as AIP.
